# Tophaceous Gout in the Pancreas: Case Reports and Review of the Literature

**DOI:** 10.1155/2022/3671627

**Published:** 2022-06-03

**Authors:** Soo Choi, Natalie Voskanian, Jeffrey Ramos, Katherine H. Y. Nguyen

**Affiliations:** ^1^University of California, San Diego, CA, USA; ^2^Scripps Memorial Hospital, San Diego, CA, USA

## Abstract

We report 3 patients who presented with abnormal pancreatic contents that were initially nondiagnostic but were eventually found to have urate crystal deposition consistent with pancreatic tophaceous gout. Our first case involved an ICU patient who had fever of unknown origin and refractory pancreatic pseudocyst. The other 2 patients presented with abdominal pain associated with a pancreatic mass which mimicked malignancy. After further investigation, we were able to identify pancreatic tophaceous gout as the diagnosis. Initiation of therapy led to resolution of pancreatitis in the first patient and resolution of abdominal pain and decrease in size of a pancreatic mass in the other 2 patients. The recognition of clinical gout involving the pancreas has important implications in the evaluation and care of these patients who are at high risk for tophaceous gout. In addition, the importance of specimen preparation that preserves crystals for viewing is discussed.

## 1. Introduction/Background

Gout is an inflammatory process initiated by macrophage ingestion of monosodium urate crystals that are needle-shaped and negatively birefringent under polarized microscopy. Tophaceous gout typically favors peripheral sites such as fingers, hands, knees, feet, toes, forearms, olecranon bursa, ears, or Achilles' tendons. Rare cases have been reported in the colon, pelvic muscles, kidneys, and spine [1–5]. However, there are very few case reports in the literature documenting tophaceous gout in the pancreas to our knowledge.

### 1.1. Case #1

A 71-year-old man with hypertension, hyperlipidemia, myocardial infarction, and distant history of gout not on treatment was brought to the hospital for slurred speech and confusion preceded by three weeks of nausea, vomiting, decreased food intake, and progressive malaise and four days of abdominal pain following escalated consumption of a bottle of vodka daily. Medications on admission were HCTZ, lisinopril, and lovastatin.

Laboratory studies revealed a significantly elevated lipase level of 6797 U/L and amylase level of 550 U/L. Other notable labs included the following: WBC 14.7 thousands/*µ*L, 80 segs and 5 bands, alkaline phosphatase 52 U/L, AST 169 U/L, ALT 71 U/L, MCV 106 fl, BUN 16 mg/dL, creatinine 12.5 mg/dL, glucose 833 mg/dL, hemoglobin A1c 5.5, creatine kinase 1986 U/L, and troponin I 0.24 ng/ml. ABG showed oxygen saturation of 89% on FiO_2_ of 60%. Blood cultures were negative throughout his hospitalization. Chest X-ray showed atelectasis and left greater than right consolidation, consistent with pneumonia. Admission CT of the abdomen and pelvis showed diffuse enlargement and edema of the pancreas and inflammatory changes of peripancreatic fat. No fluid collections, gallstones, or ductal dilatation were noted. Of interest, his lipase fell precipitously to 921 on day 2 and normalized on day 4 of hospitalization without ever being elevated again during his stay.

Despite successful treatment and resolution of his pneumonia, pleural effusions, and *C. difficile* colitis, the patient developed new onset of leukocytosis and fever up to 102 Fahrenheit. Repeat abdominal CT showed hypoperfusion of the pancreas and new development of a large fluid collection along the upper pancreatic body and tail consistent with a pseudocyst measuring 15 × 9 cm. On hospital day 22, the patient was taken to the OR for exploration and was found to have a “fully necrotic gland” and peripancreatic fluid collection. The pancreas was surgically debrided and drained, and three surgical drains were placed. Culture of the fluid contents grew out *Pseudomonas*. Another course of antibiotics was completed. The patient's surgical wound site underwent dehiscence and evisceration so he was taken back to the OR on hospital day 30 and again on day 37 for further debridement. The output from his abdominal drains was consistently greater than 100 mL daily.

On hospital day 29, the patient developed pain, swelling, and erythema of his right olecranon bursa. Bursal fluid WBC was 16,875/*µ*L with 98% neutrophils. Gram stain and culture were negative. Rheumatology was consulted on hospital day 56 for recurrent pain and swelling in the right 2^nd^ and 3^rd^ metacarpal joints and olecranon bursa. The serum uric acid level was 2.5 mg/dL. Aspiration of bursa fluid revealed chalky material positive for negatively birefringent crystals. Careful review of the surgically obtained pancreatic tissue and drain fluid confirmed the presence of birefringent monosodium crystals consistent with pancreatic tophaceous gout.

The patient's fever of 101.2 F resolved six hours after he received 20 mg Kenalog injection into the olecranon bursa. Two days later, he was placed on IV solu-medrol 40 mg, and within a day, his abdominal drain output decreased by 30% and within a week, it decreased by 90%. Patient was transferred out of ICU on day 59 and discharged after 63 days of hospitalization.

### 1.2. Case #2

A 74-year-old Asian woman with a history of CVA, hypertension, hyperlipidemia, CKD, and diuretic therapy was admitted to the hospital with two days of abdominal pain radiating to the back with normal lipase. The pain was nonpositional and not associated with eating, nausea, vomiting, or dysuria. She denied diarrhea, constipation, appetite or weight changes, fevers, chills, or night sweats.

Abdominal CT with IV contrast showed a hypoattenuating superior pancreatic mass, measuring 2.4 × 4.7 cm, and a second mass in the pancreatic head, measuring 2.2 × 1.8 cm. There was no pancreatic or biliary ductal dilatation or liver abnormality.

Her hospital course was notable for new onset polyarthritis affecting both ankles, new development of subcutaneous mass on her right ankle, fevers to 100–101 F, and an elevated white count of 12,000/*µ*L. The soft tissue mass on her ankle was aspirated, yielding 20 cc of chalky white substance that was positive for negatively birefringent crystals. The uric acid level was 10 mg/dL.

A diagnostic CT-guided FNA biopsy of the pancreatic mass revealed a necrotic and crystalline material with chronic inflammatory cells. There was no evidence of malignancy or infection. Subsequent transfer to a tertiary care hospital and endoscopic biopsy confirmed numerous needle-shaped negatively birefringent urate crystals consistent with tophaceous gout, with no evidence of malignancy or infection.

We started the patient on prednisone taper and colchicine prophylaxis with improvement of her gouty arthritis. Lasix was discontinued and allopurinol was initiated. Six months later, the pancreatic mass was unchanged on repeat CT and the patient was asymptomatic.

### 1.3. Case #3

**Figure 1:**
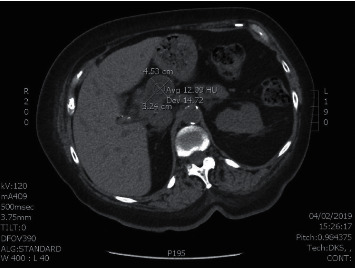
CT scan image of the abdomen without contrast showing the pancreatic mass before treatment.

A 71-year-old woman with JRA, type 2 DM, hypertension, and CKD stage 4 presented with 6 weeks of acute onset abdominal pain. CT abdomen and pelvis showed masslike area in the pancreas, measuring 4.1 × 2.7 cm (Figure 1). Endoscopic ultrasound showed 6 masses in peripancreatic, perihilar, and periportal regions. Fine-needle aspiration of the peripancreatic mass near the tail showed necrotic material with acute and chronic inflammation and rare clusters of histiocytes suggestive of granulomas. GMS and AFB stains are negative.

Rheumatology was consulted. Having previous experience with pancreatic tophaceous gout, we performed a retroactive analysis of the FNA material and confirmed the presence of needle-shaped negative birefringent crystals. The patient was started on steroid therapy and experienced significant improvement of her abdominal pain. Her uric acid was 9.0 g/dL prior to initiation of allopurinol.

**Figure 2:**
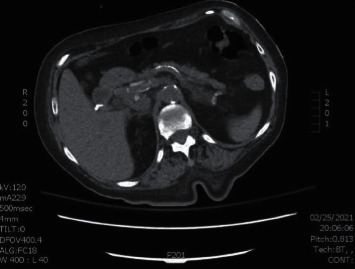
CT scan image of the abdomen without contrast showing resolution of the pancreatic mass after allopurinol therapy.

Subsequent CT abdomen performed 10 months later showed decrease in the size of the lesion, measuring 1.8 × 1.7 cm, and repeat scan in 22 months showed complete resolution of the lesion (Figure 2).

## 2. Discussion

There have only been 4 cases of pancreatic gout reported in the literature according to our search in the PubMed database for articles published in English language [6–9]. We report 3 additional cases which highlight the importance of recognizing uric acid deposition in the differential diagnosis of pancreatic lesions.

In 2002, Khanna et al. presented the first case of gouty tophi in pancreatic pseudocyst mimicking infection. In 2009, a second case of pancreatic gout was reported masquerading as neoplasm. Two additional cases of pancreatic gout mimicking neoplasm were reported in 2016 and 2019. In this case series, we report one additional case mimicking infection and two more cases mimicking malignancy. In all cases presenting as pancreatic masses, imaging findings were highly suggestive of malignancy. Biopsy of the pancreatic lesions in all of the reported cases demonstrated necrotic material and/or debris. There have been several prior case reports documenting tophaceous gout mimicking infections and malignancy in various other sites/organs [1–3]. We suggest that tophaceous gout be considered in the differential diagnosis of any soft tissue mass or fluid collection including pancreatic pseudocysts and masses.

It is important to note that 4 cases of total 7 (2 of the 4 previously reported cases and 2 of our current 3 cases) occurred in patients without a prior known history of gout. Three of the cases also had normal uric acid level at the time of diagnosis. These features may explain the paucity of pancreatic gout diagnosed reported in the literature and underscore the importance of considering pancreatic gout even in patients without a prior history of gout and with normal uric acid level. We hypothesize that pancreatic gout may explain cases of pancreatitis previously labeled idiopathic. It should be noted that the usual method by which surgical tissue is prepped using aqueous fixations, especially with use of formalin, often results in dissolution of the urate crystals. We suspect that the diagnosis of pancreatic gout may have been missed due to the fixation and preservation process dissolving the crystals. We suggest that any patient with a history of gout who is undergoing surgery or biopsy of the pancreas in the setting of pancreatitis have additional specimens sent to look for urate crystals in a nonaqueous solution [10].

All of the previously reported cases of pancreatic gout were either asymptomatic or only associated with mild nonspecific abdominal pain. Our case ^#^1 is the first case documenting pancreatic gout associated with fever and leukocytosis. Our case demonstrates that pancreatic gout can explain fever of unknown origin in a patient with pancreatic fluid collection. In this case, refractory fever after >50 days of ICU admission and several courses of antibiotics and surgeries resolved within six hours of initiation of steroid therapy and his pancreatic drain output decreased by 30% within 1 day and by 90% within 1 week of starting solu-medrol. In all of the reported cases, urate-lowering therapy was associated with clinical improvement. In cases associated with pancreatic lesions, the lesions remained stable at 6 months in 2 cases and improved or resolved at 8–18 months in 3 cases. These cases highlight the importance of exploring the possibility of pancreatic gout and considering steroid and urate-lowering therapy in patients with pancreatic fluid collections/lesions.

We have shown that tophaceous gout in the pancreas can mimic pseudocyst, pancreatic abscess, or malignancy. We hope that this case series expands awareness of pancreatic gout and encourages additional evaluation in future patients who present with pancreatic abnormalities and gout risk factors. Making the appropriate diagnosis is crucial in preventing unnecessary studies, interventions (i.e., antibiotics, chemotherapy), surgeries, and prolonged hospitalization and results in overall improved patient care.

## Data Availability

The information can be provided upon request to the corresponding author.
